# Research on the relationship between gaze anxiety, social media disorder, and perceived discrimination in medical students

**DOI:** 10.1038/s41598-025-05705-7

**Published:** 2025-07-01

**Authors:** Yanan Zheng, Die Hu

**Affiliations:** 1 Department of Psychology, Gannan Medical University, Ganzhou City, Jiangxi Province China; 2 Medical History in Central Soviet Area and Healthy China Research Center, Gannan Medical University, Ganzhou City, Jiangxi Province China; 3 Nursing Department, Xuzhou Renci Hospital, Xuzhou City, Jiangsu Province China

**Keywords:** Medical students, Gaze anxiety, Social media disorder, Perceived discrimination, Human behaviour, Health care

## Abstract

To explore the current situation of gaze anxiety and its relation with social media disorder and perceived discrimination in medical students, this study aimed to provide insights for the reform of medical communication education. A cross-sectional design was adopted, 811 medical students were selected, and they were investigated with the Chinese version of gaze anxiety rating scale, the Chinese short version of social media disorder scale and perceived discrimination questionnaire in May 2022. The results revealed that medical students’ gaze anxiety was the highest in the daily dimension, and there was a statistically significant difference among different grades (*F* = 13.063, *p* < 0.001); gaze anxiety was positively correlated with social media disorder (*r* = 0.504, *p* < 0.01) and perceived discrimination (*r* = 0.505, *p* < 0.01); social media disorder has a direct positive effect on medical students’ gaze anxiety and may also indirectly influence it through perceived discrimination. The conclusion was that perceived discrimination can play a partial mediating role between gaze anxiety and social media disorder among medical students.

## Introduction

The concept of gaze anxiety is rather complex. It falls under the domain of social anxiety and refers to the avoidance of eye contact exhibited by individuals with social anxiety disorder and poor interpersonal communication skills^1,2^as gaze can convey competitive, dominant, and intimate-related information^3,4^. Building upon the phenomenon of gaze avoidance, individuals often worry about the potential offense or the corresponding negative evaluations caused by diverting their gaze. Expanding upon this foundation, Schneier further proposed the concept of gaze anxiety, which encompasses both gaze avoidance behavior and the accompanying subjective experience of anxiety^1^.

Social anxiety is a common interpersonal communication issue among college students^5^. However, medical students represent a unique group within this population. Due to the specific demands of their profession and the need for future competency in their respective roles, medical students are expected to possess not only professional skills and a strong work ethic but also effective communication abilities^6^. Thus, their communication skills and social anxiety problems merit further attention from medical education circles. Existing studies have consistently shown that medical students have a high prevalence of social anxiety^7–9^ and generally low levels of communication competence^10^. However, most of these studies focus on medical students’ attitudes towards communication skills learning and how to conduct doctor-patient communication education^11–13^or specific components of interpersonal communication abilities such as empathy, communication apprehension, and interpersonal efficacy^14–16^. Limited attention has been given to gaze anxiety. Current research on gaze anxiety primarily targets patients^1^ or non-medical college students^17^with little focus on medical students as a distinct population. If medical students experience gaze anxiety, it could potentially have a negative impact on their future medical practice education and doctor-patient communication in clinical. For instance, eye contact with patients may lead to heightened nervousness and hinder effective communication. Consequently, it is of great importance to explore the current status and influencing factors of gaze anxiety among medical students and targetedly implement psychological health education and doctor-patient communication training to enhance their communication abilities. This endeavor holds significant value and importance in cultivating healthcare professionals with a strong professional ethos, and it also contributes to the theoretical advancement of research in the field of interpersonal communication.

Social media disorder refers to the inability of individuals to integrate into normal social activities due to excessive use of virtual social media platforms such as WeChat, Weibo, QQ, and online games. Reducing the time spent on social media can lead to cognitive and emotional issues, affecting real-life interpersonal communication^18^. Numerous studies have indicated a positive correlation between excessive smartphone use and social anxiety^19,20^. Based on these, hypothesis 1 is proposed in this study: social media disorder positively predicts gaze anxiety among medical students.

Perceived discrimination refers to the subjective perception of unfair treatment by others based on one’s identity and behavior^21^. According to Social Information Processing Theory^22^individuals’ interpretations of information shape their subsequent attitudes and behaviors. For individuals who experience social difficulties due to excessive internet use, their discomfort in social interactions may lead to a more negative perception of the surrounding world. Similarly, based on this theory, individuals with perceived discrimination are more likely to interpret the outcomes of eye contact negatively, thereby experiencing gaze anxiety. In addition, relevant research has shown a connection between perceived discrimination and both smartphone dependency and interpersonal communication^23–25^. Additionally, perceived discrimination is significantly positively correlated with social anxiety^26^. Based on these, hypothesis 2 is proposed in this study: perceived discrimination mediates the relationship between social media disorder and gaze anxiety among medical students.

In summary, existing research on gaze anxiety has primarily focused on patients and general college student populations, with limited attention to medical students. Moreover, few studies to date have explored the underlying mechanisms involving social media disorder and perceived discrimination. To investigate the current status of gaze anxiety among medical students and its relationship with perceived discrimination and social media disorder, a questionnaire survey was conducted in May 2022 among medical students who from a Chinese medical university. The details are reported as follows.

## Materials and methods

### Participants

A cross-sectional study was conducted. A total of 827 students from the clinical medicine major at various levels, including freshmen to seniors, were selected using cluster sampling from Gannan Medical University. Due to the fifth-year college students being in the clinical practice stage, factors influencing gaze anxiety are more complex due to direct exposure to doctor-patient interactions. Moreover, this study focused on students in the theoretical learning stage, where interventions may be more effective in alleviating gaze anxiety and related interpersonal communication stress. Therefore, the level of gaze anxiety among graduating seniors(the fifth-year college students) is of limited significance to this survey and they were not included in this study. Questionnaires were distributed to students by members of the research team during the break time, and on-site collection was conducted. A total of 827 questionnaires were distributed, of which 820 were recovered, making a recovery rate of 99.15%; 811 were valid, making a validity rate of 98.07%. Given the participants shared a similar academic background, with primary differences observed in gender and grade level, and in line with the objectives of this study, only gender and grade were included as sociodemographic variables. Among the participants, there were 402 males and 409 females. The distribution across grade levels was as follows: 213 freshmen, 210 sophomores, 222 juniors, and 166 seniors. Inclusion criteria: students who consent to participate in this study with full knowledge of its content and purpose; Exclusion criteria: participants who did not complete all questionnaire items.The study was conducted according to the guidelines of the Declaration of Helsinki and approved by Biomedical Research Ethics Committee of Gannan Medical University(Protocol code: Ref number 2022361/29 January 2022).

### Questionnaires

#### The Chinese version of gaze anxiety rating scale

The gaze anxiety level among medical students was assessed using the gaze anxiety rating scale developed by Langer, J. K. et al.^27^ and later adapted by Li, X. et al.^17^ for Chinese participants based on Chinese culture and language. The scale consists of 14 items, requiring participants to evaluate their levels of fear based on specific scenarios (e.g., being introduced to others). It includes three dimensions: performance (7 items, assessing the level of gaze anxiety due to concerns about personal abilities), public speaking (3 items, assessing the level of gaze anxiety during public speaking), and daily life (4 items, assessing the level of gaze anxiety in daily activities). The scale uses a 4-point scoring system ranging from 0 (not at all) to 3 (very much). The total score of the scale, as well as the scores for each dimension, was calculated by summing the scores of individual items. The total score and scores for each dimension indicate the severity of gaze anxiety. In this study, the Cronbach’s *α* of this scale was 0.901.

#### The Chinese short version of social media disorder scale

The Chinese short version of social media disorder scale was used to assess the level of social media disorder among medical students. The scale was originally developed by Regina, J. et al.,^28^ and Zhang, L., et al. modified this scale to make it suitable for evaluating Chinese undergraduates based on Chinese culture and language^29^. The scale consists of 9 items, including two dimensions: real-life social failure and real-life social conflict. Participants rate each item on a 5-point scale ranging from 1 (completely disagree) to 5 (completely agree). The total score reflects the severity of social media disorder. In this study, the Cronbach’s *α* of this scale was 0.859.

#### Perceived discrimination questionnaire

The personal perceived discrimination subscale was adopted in this study to assess the level of perceived discrimination among medical students, which from the perceived discrimination questionnaire developed by Shen, J. et al.^30^. This scale has been widely applied in studies measuring perceived discrimination among university students^31,32^.The questionnaire consists of 3 items, and participants rate each item on a 5-point scale ranging from 1 (completely disagree) to 5 (completely agree). The total score represents the intensity of perceived discrimination. In this study, the Cronbach’s *α* of this questionnaire was 0.857, and the split-half reliability was 0.869. The composite reliability (CR) was 0.859 (> 0.7), and the average variance extracted (AVE) was 0.669 (> 0.5). All standardized factor loadings were greater than 0.7 (q1 = 0.831, q2 = 0.776, q3 = 0.845). According to item response theory(IRI), the discrimination parameters (*a*-parameters) for all items were above 1.7^33^ (q1 = 3.29, q2 = 2.84, q3 = 4.18), indicating that the items effectively distinguished between respondents with different levels of perceived discrimination. The difficulty parameters (*b*-parameters) were appropriately distributed, covering a broad range of latent trait levels among participants.

### Statistical analysis

Data management and analysis were performed using SPSS 23.0. Independent-samples *t* test and analysis of variance (ANOVA) were used to examine differences in gaze anxiety levels among different groups, with Bonferroni test applied for multiple comparisons. Pearson correlation analysis and the PROCESS macro (Model 4) were used to test the mediating effect. Statistical significance was defined as *p* < 0.05.

## Results

### Common method bias test

All data in this study were collected through self-reported questionnaires from medical students. To assess the presence of common method bias, Harman’s single-factor test was conducted prior to data analysis. The results showed that five factors had eigenvalues greater than 1, and the first factor accounted for 35.13% of the total variance, which is below the critical threshold of 40%. This indicates that common method bias was not a serious concern in this study.

### General situation of gaze anxiety among medical students

Among 811 medical students, the score of the gaze anxiety rating scale was 11.76 ± 7.20. The average scores for the performance, public speaking, and daily life dimensions were 0.86, 0.57, and 1.01 per item, respectively. There were no statistically significant differences in gaze anxiety scores between different genders, while significant differences were found among different grades. Please refer to Table 1 for detailed information.

**Table 1:** Comparison of gaze anxiety rating scores among medical students with different demographic characteristics.

		Gaze Anxiety			Social Medial Disorder			Perceived Discrimination			
Factors		M ± SD	*t*(*F*)	(Partial) *η*^2^	M ± SD	*t*(*F*)	(Partial) *η*^2^	M ± SD	*t*(*F*)	(Partial) *η*^2^	
Gender	Male(*n* = 402)	12.08 ± 7.56	1.202	0.002	18.47 ± 6.61	2.499*	0.008	5.78 ± 2.38	2.512*	0.006	
	Female(*n* = 409)	11.47 ± 6.83			17.39 ± 5.61			5.45 ± 1.91			
Grade	Freshmen(*n* = 213)	12.57 ± 7.73	13.063***	0.046	17.73 ± 6.19	5.844**	0.021	5.56 ± 2.53	7.571***	0.027	
	Sophomores(*n* = 210)	10.81 ± 6.96			18.17 ± 5.76			5.63 ± 2.03			
	Juniors(*n* = 222)	10.29 ± 7.18			16.79 ± 6.17			5.19 ± 1.99			
	Seniors(*n* = 166)	14.25 ± 5.96			19.37 ± 6.25			6.23 ± 1.87			

①Bonferroni test: seniors> juniors***, seniors> sophomores***, freshmen> juniors**, freshmen> sophomores*(Based on the research objective, multiple comparisons were conducted only on the differences in gaze anxiety scores across different academic years).

②(Partial) η²: an effect size measure used in t-tests and analysis of variance (ANOVA) to quantify the proportion of variance in the dependent variable explained by a particular factor.

### Correlation analysis of gaze anxiety, social media disorder, and perceived discrimination among medical students

The correlation analysis showed significant positive correlations between gaze anxiety and social media disorder, gaze anxiety and perceived discrimination, and social media disorder and perceived discrimination. Refer to Table 2 for details.

**Table 2:** Correlation analysis of gaze anxiety, social media disorder, and perceived discrimination.

	M ± SD	Gaze anxiety	Perceived discrimination	Social media disorder
Gaze anxiety	11.76 ± 7.20			
Perceived discrimination	17.92 ± 6.14	0.505**		
Social media disorder	5.61 ± 2.16	0.504**	0.583**	

### The mediating role of perceived discrimination between gaze anxiety and social media disorder among medical students

**Fig. 1:**
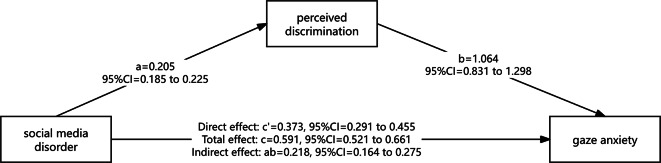
The mediating role of perceived discrimination in the relationship between gaze anxiety and social media disorder among medical students.

The correlation analysis revealed a statistically significant relationship among gaze anxiety, social media disorder, and perceived discrimination among medical students. To further explore the relationship among these variables, mediation analysis was conducted using the PROCESS macro (Model 4). Gaze anxiety was set as the dependent variable, social media disorder as the independent variable, and perceived discrimination as the mediating variable. According to the Bootstrap method of Hayes^34^ “A sample size of 5000 was chosen with a confidence interval of 95%”, was used to test whether there was a mediating effect between the three variables. The results showed the mediating interval between social media disorder and gaze anxiety(LLCI = 0.164, ULCI = 0.275) did not contain 0, suggesting that perceived discrimination had a significant mediating effect between social media disorder and gaze anxiety. Furthermore, after controlling for the mediator, social media disorder had a significant effect on the gaze anxiety, and the interval (LLCI = 0.291, ULCI = 0.455) did not contain 0, suggesting that perceived discrimination plays a partial mediating role in the relationship between social media disorder and gaze anxiety. The mediating effect accounted for 36.89% of the total effect. Detailed results are presented in Fig. 1.

## Discussion

### Major manifestations of gaze anxiety among medical students and notable variances across different grades

The survey results revealed that the scores of gaze anxiety among medical students fell below the midpoint of the scale and primarily manifest in daily gaze anxiety scenarios, such as discussing issues with several individuals, socializing with cashiers while shopping, and listening to others’ speeches. The causes could be attributed to the nature of medical specialties, which come with substantial academic pressure and demand extensive time for assignment completion and laboratory participation, which might inhibit regular social activities to some extent. The findings suggest the significance of fostering daily campus activities and creating communicative environments in medical colleges. The study also revealed that medical students had the lowest level of anxiety during public speaking. The probable reason for this is not necessarily the proficiency of medical students in public speaking but rather the lack of experience in public speaking, resulting in a deficiency of gaze anxiety experience during such situations. This lack of experience contributes to lower anxiety ratings compared to the more familiar gaze anxiety experienced during routine social interactions. Although the future careers of medical students may not be significantly related to public speaking, scenarios such as case discussions, patient briefings to families do require some public speaking skills. Therefore, medical colleges should consider incorporating solo presentations, reports, and other teaching methods to increase students’ social interactions and reduce gaze anxiety.

In terms of grade differences, the survey results revealed that gaze anxiety was more severe among freshmen and senior students. The reasons could potentially be attributed to freshmen being in the adaptation phase between interpersonal relationships and the environment, while seniors face heavy internship, employment, and postgraduate examination pressures. Particularly, gaze anxiety brought by doctor-patient communication becomes more pronounced. The findings suggest that medical colleges should launch necessary social training for different grades, such as assisting freshmen in better adapting to their environment during their first year, conducting necessary doctor-patient communication skill training in the later stages, and helping medical students adapt to the future medical work environment through “early clinic” and other approaches. However, this study did not discover any differences in gaze anxiety between different genders among medical students. The result is similar with findings by Zhang, H. et al.^35^ and Zhu, Y. et al.^36^who reported no significant gender differences in social anxiety among Chinese students, contrasting with Domes, G. et al.^37^ who discovered higher gaze anxiety in German females. One possible explanation for this discrepancy lies in cultural differences. In collectivist cultures like China, maintaining social harmony is a shared social value that often takes precedence over individual emotional expression^38^. Both males and females may therefore be more inclined to suppress negative emotions in order to preserve interpersonal relationships, which could reduce gender-based differences in anxiety expression. Additionally, among medical students, academic pressure and the stressful work environment may exert a more pronounced influence on gaze anxiety than differences in gender roles^39,40^.

### Correlation between gaze anxiety, social media disorder, and perceived discrimination among medical students

The correlation analysis results of gaze anxiety, social media disorder, and perceived discrimination among medical students suggested that the more severe the social media disorder is among medical students, leading to increased detachment from real-life social interactions, impacting real-world activities, and fostering perceptions of being looked down upon or treated unfairly, the more pronounced the level of gaze anxiety becomes. At present, there are limited research findings on gaze anxiety and social media disorder. However, this study’s findings are similiar with those of Teng, X. et al., who concluded that “there is a close relationship between social network addiction and social anxiety in college students”^41^Mao, Y. et al., who found that “there is a connection between loneliness and perceived discrimination among college students”^42^and Yang, X. et al., who discovered that “there is a strong correlation between mobile phone dependence and social anxiety among college students”^19^. It suggests that an individual’s interpersonal communication may be closely related to overuse of the internet and perceived prejudice from others. Social media disorder reflects the extent to which individuals’ real-life social interactions are impacted due to excessive use of online social media, such as alleviating negative emotions through online social media and overuse of online social media affecting other social activities. Medical students with more severe social media disorder tend to neglect real-life social interactions, thereby exacerbating gaze anxiety due to “unfamiliarity.” On the other hand, students with more severe perceived discrimination tend to interpret others’ actions from an “unfair” perspective, impacting interpersonal trust levels, thereby easily inducing anxiety during social activities and leading to gaze anxiety. The findings suggest that medical colleges could consider reinforcing the dissemination of scientific knowledge on the use of social media, effectively managing the use of WeChat, QQ, and other social media platforms, creating a favorable campus atmosphere, and enhancing a sense of responsibility to improve the humanistic spirit among medical students, reduce their level of perceived discrimination, and hence reduce gaze anxiety and effectively enhance their communicative skills.

### Partial mediating effect of perceived discrimination among medical students between gaze anxiety and social media disorder

The results of the mediation analysis revealed that social media disorder among medical students could both directly influence the level of gaze anxiety and exert a mediating effect on gaze anxiety through perceived discrimination. Individuals with gaze anxiety often worry about negative evaluations brought about by gazing during interactions, such as exposing their thoughts and feelings of nervousness, which is associated with social anxiety. Perceived discrimination, which results from individuals feeling prejudiced or unfairly treated by others due to their behavior patterns, can affect interpersonal communication and social abilities to a certain extent due to the sensitivity regarding others’ “evaluations”. Social media disorder does not solely entail excessive use of social networking media such as WeChat by individuals, but also involve assessing the extent of the impact on real-life social interactions caused by excessive use of online social media. According to social information processing theory, past experiences shape individuals’ interpretations of social information, thereby influencing their subsequent attitudes and behaviors. Students with severe social media disorder often experience social difficulties due to excessive online engagement, which fosters negative perceptions of social interactions and leads to social avoidance. Their limited real-life interaction makes it harder for them to adapt to offline interpersonal contexts, particularly in situations requiring self-expression, such as public speaking or presenting opinions. The stark contrast between online and offline communication—such as fears of being unclear or unintentionally offensive—may trigger social anxiety and exacerbate gaze anxiety. Furthermore, according to social information processing theory, individuals with internet addiction-related social impairments often develop negative interpretations of social experiences. They are more likely to perceive others’ behaviors as biased or unfair, leading to heightened perceived discrimination. This tendency to suspect hostility from others increases interpersonal sensitivity and further exacerbates gaze anxiety. Generally, research on perceived discrimination often focuses on disadvantaged groups such as financially challenged students and disabled college students^31,43^while relatively little attention has been paid to individuals outside these groups However, it should be noted that even individuals who are not part of traditionally disadvantaged populations might develop perceived discrimination, believing that others are prejudiced against them due to reasons such as lack of social abilities. Moreover, medical students with more severe social media disorder often have an excessive dependence on online social networking, making it hard to simply require them to reduce the use of online social media. In addition to communication skills training, on one hand, targeted interventions should include mindfulness practices and Internet-Based Cognitive Behavioral Therapy to alleviate social anxiety, rather than simply directly restricting online activity^44^. A growing body of research has shown that mindfulness practice^45^ helps reduce smartphone dependency^46,47^psychological distress^48^and anxiety among individuals with internet addiction^49^. Internet-Based Cognitive Behavioral Therapy^50,51^ offers a digital approach that helps reduce social anxiety in individuals who are unwilling to engage in face-to-face interactions. In addition, digital literacy training^52^ should be provided to help individuals develop a proper understanding of the internet, reduce excessive reliance on it, and thereby alleviate social difficulties caused by internet overuse. Meanwhile, for medical students, excessive academic pressure is one of the key factors contributing to their social media use disorder^53,54^. Therefore, training in time and energy management should be provided to help reduce their dependence on social media, thereby alleviating gaze anxiety. On the other hand, boosting self-confidence, setting clear goals, and providing education on professional ethics may help medical students develop a fairer understanding of the world^55,56^reducing their subjective perception of being “discriminated against.” Workshops on discrimination^57^ can also help reduce both the subjective and objective experiences of discrimination. These approaches can effectively reduce gaze anxiety among medical students, enhance their communication abilities, and maintain their psychological well-being.

## Conclusion

The results of this study indicated that the level of gaze anxiety among medical students is relatively low to moderate, mainly manifested as daily gaze anxiety. Social media disorder not only directly affects the level of gaze anxiety among medical students but also has an indirect effect through the mediating role of perceived discrimination. This suggests that medical colleges should not only focus on communication skills training for students with social media disorders but also address helping students reduce their subjective feeling of “being prejudiced”.

The innovation of this study lies in its unique focus on gaze anxiety among medical students, an area that has received comparatively less attention, especially from the perspectives of social media disorder and perceived discrimination. While existing studies on gaze anxiety have mainly concentrated on patients or general university students, this research explores the issue from the perspective of medical students, offering new insights into the contributing factors of gaze anxiety. Additionally, by investigating gaze anxiety in medical students, this study can help medical schools enhance students’ communication skills, better preparing them to meet the demands of their future professional roles in clinical settings.

The limitations of this study mainly lie in the fact that the investigation into the current level of gaze anxiety among medical students was limited to one medical school in China, and the sample size was relatively small, which may not represent a broader range of students in China. In addition, the research findings were not compared with the gaze anxiety levels of medical students from other countries, indicating a limited perspective in the study. Moreover, the study employed a cross-sectional design, which restricts the ability to draw causal inferences. Additionally, the study excluded fifth-year medical students. This exclusion may have limited the comprehensiveness of the findings. Therefore, future research on the current level and influencing factors of gaze anxiety among medical students could conduct larger-scale investigations with a wider sample in China and place the research findings in an international context. Future research could adopt a longitudinal design to better explore causal relationships between gaze anxiety and its influencing factors and consider including and comparing both intern and non-intern medical students to provide a more complete understanding of gaze anxiety throughout different stages of medical training.

## Data Availability

The data used and analysed during the current study available from the corresponding author on reasonable request.
